# The Role of Breath Analysis in the Non-Invasive Early Diagnosis of Malignant Pleural Mesothelioma (MPM) and the Management of At-Risk Individuals

**DOI:** 10.3390/molecules30193922

**Published:** 2025-09-29

**Authors:** Marirosa Nisi, Alessia Di Gilio, Jolanda Palmisani, Niccolò Varesano, Domenico Galetta, Annamaria Catino, Gianluigi de Gennaro

**Affiliations:** 1 Department of Biosciences, Biotechnologies and Environment, University of Bari Aldo Moro, 70126 Bari, Italy; marirosa.nisi@uniba.it (M.N.); gianluigi.degennaro@uniba.it (G.d.G.); 2 Apulian Regional Centre for the Breath Analysis, Istituto Tumori ‘Giovanni Paolo II’, 70126 Bari, Italy; galetta@oncologico.bari.it (D.G.); annamaria.catino@gmail.com (A.C.); 3 Thoracic Oncology Unit, Istituto Tumori ‘Giovanni Paolo II’, 70124 Bari, Italy; niccovaresano@gmail.com

**Keywords:** breath analysis, VOCs, diagnosis, malignant pleural mesothelioma, biomarkers, MPM follow-up, at-risk asbestos exposed subjects

## Abstract

Malignant pleural mesothelioma (MPM) is a rare and aggressive malignancy associated with occupational or environmental exposure to asbestos. Effective management of MPM remains challenging due to its prolonged latency period and the typically late onset of clinical symptoms. Accordingly, there is an increasing demand for the implementation of reliable, non-invasive, and data-driven diagnostic strategies within large-scale screening programs. In this context, the chemical profiling of volatile organic compounds (VOCs) in exhaled breath has recently gained recognition as a promising and non-invasive approach for the early detection of cancer, including MPM. Therefore, in this cross-sectional observational study, an overall number of 125 individuals, including 64 MPM patients and 61 healthy controls (HC), were enrolled. End-tidal breath fraction (EXP) was collected directly onto two-bed adsorbent cartridges by an automated sampling system and analyzed by thermal desorption–gas chromatography–mass spectrometry (TD-GC/MS). A machine learning approach based on a random forest (RF) algorithm and trained using a 10-fold cross-validation framework was applied to experimental data, yielding remarkable results (AUC = 86%). Fifteen VOCs reflecting key metabolic alterations characteristic of MPM pathophysiology were found to be able to discriminate between MPM and HC. Moreover, twenty breath samples from asymptomatic former asbestos-exposed (AEx) and eight MPM patients during follow-up (FUMPM) were exploratively analyzed, processed, and tested as blinded samples by the validated statistical method. Good agreement was found between model output and clinical information obtained by CT. These findings underscore the potential of breath VOC analysis as a non-invasive diagnostic approach for MPM and support its feasibility for longitudinal patient and at-risk subjects monitoring.

## 1. Introduction

Malignant pleural mesothelioma (MPM) is a rare but highly aggressive neoplasm of the pleura, the membrane lining the lungs. A complex interaction of factors, including genetic mutations, epigenetic alterations, and dysregulation of cell signaling pathways, plays a role in its pathogenesis, development, and progression [1]. Mesothelioma has been associated with exposure to asbestos, a material widely used in construction, shipbuilding, and industry thanks to its durability, insulating properties, and resistance to chemicals and fire [2,3]. In fact, the widespread use of asbestos since the 1920s, which peaked between the 1970s and 1980s, has been linked to a substantial increase in mesothelioma incidence. Moreover, although asbestos use has been banned in Europe since 1999 and worldwide production declined between 1980 and 2010, to date, it remains a serious human health hazard [4,5,6]. In 2022, the Global Cancer Observatory reported 30,633 global cases of mesothelioma, with higher incidence rates in men living in Northern Europe, especially in developed regions where occupational exposure in historically asbestos-heavy industries was more widespread [7]. In addition to pleural mesothelioma, asbestos fibers are strongly linked to malignancies such as lung, laryngeal, and ovarian carcinoma as well as benign diseases such as asbestosis, pleural plaques, pleural thickening, and effusion [8,9]. Although asbestos is undeniably involved in mesothelioma causation, the exact pathogenetic mechanisms underlying the disease remain unclear. Human mesothelial cells, although highly sensitive to asbestos cytotoxicity, can become cancerous through a variety of pathogenetic processes. It has been hypothesized that asbestos fibers repeatedly scratch the mesothelial surface, causing prolonged cycles of damage, repair, and local inflammation. Moreover, asbestos can generate reactive oxygen species (ROS) and reactive nitrogen species (RNS), causing DNA damage and thereby contributing to MPM development. Finally, Faux et al. [10] showed that asbestos can stimulate the production of pro-inflammatory cytokines (such as TNF-α and IL-1β) and growth factors (like TGF-β and VEGF), contributing to a microenvironment favorable to tumor development. Additionally, asbestos exposure can activate transcription factors, including NF-κB and AP-1, which regulate gene expression involved in cell proliferation and, thus, tumor progression [10,11].

MPM is usually detected 20 to 40 years after exposure to asbestos, following a long latency period between exposure and diagnosis [12]. Currently, average patient survival is less than one year, and the five-year survival rate remains below 5% due to the aggressive nature of the cancer, late diagnosis, and current treatment options with limited efficacy. Therefore, considering that the MPM diagnosis is to date based on imaging and biopsy, there is an urgent need for sensitive and non-invasive screening tools to enhance early diagnosis and optimize therapeutic management of MPM [5,13]. An ideal option could be the identification of potential biomarkers capable of discriminating MPM patients from healthy individuals and predicting early signs of malignant transformation in asbestos-exposed individuals [14]. Recent studies show that the analysis of volatile organic compounds (VOCs) in exhaled breath can provide a useful tool for the diagnosis of patients with neoplasms and asbestos-related diseases [15]. In fact, breath analysis represents a less invasive alternative to computed tomography scan (CT scan) and biopsy and enables easier sample collection than blood or urine analysis.

Moreover, it is now well known that the volatile fraction of human breath contains thousands of low-molecular-weight compounds which are the final products of the countless metabolic and cellular processes continuously occurring in our bodies at the tissue level [5,6,16,17]. Therefore, disease-related metabolic and biochemical alterations may result in the production of specific VOCs that enter the bloodstream, reach the lungs, and are subsequently exhaled via diffusion across the alveolar-capillary membrane [16]. Volatile organic compounds detected in breath and linked to an oncological pathology may result from several mechanisms. Firstly, oxidative stress induces lipid peroxidation, resulting in cell and mitochondrial membrane damage and, thus, alkane, alkene, and aldehyde formation. Secondly, exposure to environmental carcinogens and ROS accumulation causes the overexpression of cytochrome P450 enzymes, a common factor in the onset and progression of several cancers. In addition, cancer metabolic reprogramming, characterized by a reduction in oxidative phosphorylation in favor of aerobic glycolysis (Warburg effect), and molecular alterations in oncogenes and oncosuppressor genes, further contribute to VOCs production [17]. Thirdly, the AMP-activated protein kinase (AMPK) acts as a cell energy sensor. In fact, when cell energy decreases, such as in neoplasm, AMPK brings energy back to normal levels, blocking energy-consuming processes and activating energy-producing ones, like fat burning. Moreover, AMPK is activated by another protein, called LKB1, which has a role in controlling cell growth and acts as a tumor suppressor. Therefore, changes in AMPK activation and subsequent metabolic pathways linked to cancer may affect qualitatively and quantitatively the formation and release of metabolites as VOCs in the blood and other biological matrices such as breath [18].

Until now, few studies have explored the potential of breath analysis, in terms of volatile organic compounds detection in human breath, as a method for the early diagnosis of malignant pleural mesothelioma. Studies focused on the use of advanced technological solutions that are easy to use and able to perform real-time analysis of VOCs in human breath. For example, multicapillary column ion mobility spectrometry (MCC/IMS) and sensor arrays, also known as e-nose, were used for real-time monitoring of VOCs, opening the way to new possibilities for non-invasive and timely diagnosis of diseases such as MPM [15,19,20,21,22,23,24,25]. However, although the potential of such innovative approaches for the early and non-invasive diagnosis of MPM was highlighted, the cohorts included in these studies were limited, affecting the generalizability and reproducibility of the obtained results. Lamote et al. [19] analyzed by using MCC/IMS, breath samples exhaled from 23 MPM patients, 20 asbestos-exposed persons (AEx), and 21 healthy controls (HC) and obtained a sensitivity of 87% in discriminating MPM patients from both AEx and HC controls [19]. Dragonieri et al. [22] used an electronic nose (Cyranose 320) to analyze breath samples exhaled from 13 patients with histologically confirmed MPM, 13 long-term AEx, and 13 HC. The authors showed that the breath prints of MPM patients were significantly different (with a sensitivity of 92.3%) from those obtained by the analysis of breath samples collected from asbestos-exposed subjects [22]. In the study conducted by Chapman et al. [23], 20 patients with MPM, 18 subjects with asbestos-related diseases, and 42 healthy controls were recruited in a cross-sectional study. Using the carbon polymer array (CPA) electronic nose, the authors were able to distinguish between MPM patients and controls with a sensitivity of 90% [23]. On the other hand, it has been well documented that gas chromatography-mass spectrometry, considered the gold standard in breath analysis, shows the highest accuracy in distinguishing malignant pleural mesothelioma (MPM) patients from at-risk asbestos-exposed individuals [24,26,27]. De Gennaro et al. [28] discriminated 13 MPM patients from 13 HC controls and 13 AEx persons with an accuracy of 97.4% considering variables such as cyclopentane, cyclohexane, dodecane, dimethyl nonane, limonene, and β-pinene [28]. Lamote et al. [21] enrolled 16 HC, 19 asymptomatic asbestos-exposed individuals (AEx), 15 with benign asbestos-related diseases (ARD), and 14 patients with MPM. The authors achieved an accuracy of 97% in the discrimination between MPM and AEx, and of 94% grouping AEx and ARD [21]. Similar results were obtained in our previous study, conducted enrolling 14 MPM and 20 HC. An accuracy of 93% was obtained in discrimination between MPM and HC by using ketones, alkanes, methylated derivatives, and hydrocarbons as biomarkers [5].

Taking into account the limitations of previously published studies, the main aim of this work is to confirm, in a larger study population, the strengths of breath analysis for the early diagnosis of MPM. Moreover, based on the results obtained, we explored the clinical utility of breath analysis and, in particular, of the developed approach for both the screening of asbestos-exposed individuals and the longitudinal assessment of MPM during follow-up, with the aim of identifying metabolic alterations associated with disease onset, progression, and recurrence as well as establishing a non-invasive method for disease monitoring and treatment response evaluation.

## 2. Results

**Figure 1:**
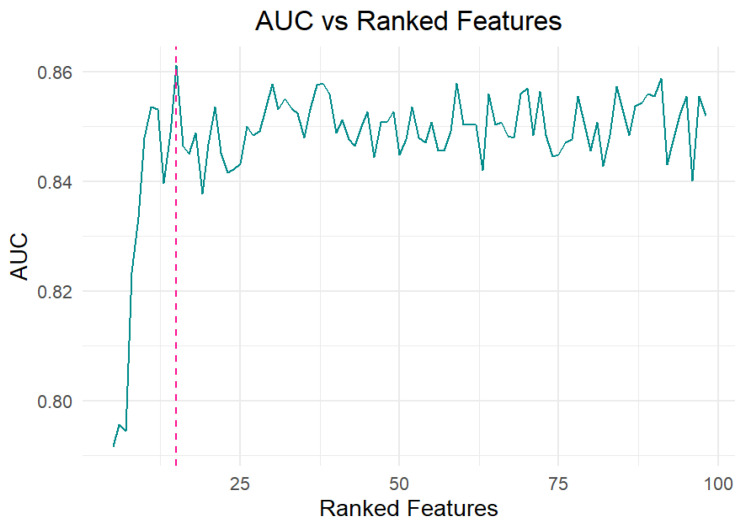
The Area Under the Curve (AUC) values reflect the performance of the Ranked Features (RF) model as a function of the number of top-ranked features included.

In the present study, an overall number of 125 AA and 125 breath samples collected from healthy subjects (HC) and patients affected by malignant pleural mesothelioma (MPM) were analyzed using the TD-GC/MS technique. The analysis of the obtained chromatograms enabled the identification of 106 different volatile organic compounds (VOCs), characterized by retention times between 3.6 and 40.1 min and main mass-to-charge ratios (m/z) between 39 and 168 m/z. Among them, 98 VOCs were detected in over 90% of the breath samples and were not linked to device components and materials contamination. The data matrix, including 98 VOCs (variables) in 250 samples, was processed by nonparametric Wilcoxon/Kruskal–Wallis tests (R version 3.6.4), allowing the identification of the most weighted variables (endogenous VOCs) able to discriminate among breath samples and ambient air (AA). More specifically, the abundances of 27 VOCs in breath samples were found to be significantly different with respect to those detected in ambient air samples (AA). In order to identify specific VOC pattern associated with disease and able to discriminate between HC and MPM, a random forest (RF) analysis was applied to three major categories of datasets: (i) 27 endogenous VOCs obtained by applying a non parametric test, (ii) across all 98 VOCs detected in breath samples (abundance_exhaled_), or (iii) across all 98 ambient-subtracted VOCs obtained by subtracting the abundance of ambient air from exhaled breath for each VOC (Δabundance = abundance_exhaled_ − abundance_ambient_). In all three cases, the model was optimized to enhance predictive performance through the implementation of a 10-fold cross-validation approach. No significant difference in terms of model classification performance was observed in the three considered cases, probably because the broader spectrum of considered VOCs provides important and unfiltered information. Although this broader spectrum may increase environmental noise, it appears to be effectively managed by the random forest algorithm. In addition, ambient-subtracted data offer an accurate metabolic or endogenous VOC profile, but do not take into account weaker yet potentially informative signals [29]. Therefore, since the statistical model applied to unsubtracted breath data (98 VOCs for 125 breath samples) demonstrated slightly superior classification performance, only the results from this statistical approach are reported below. As shown in Figure 1, analysis of the area under the curve (AUC) as a function of the number of features selected by the model revealed that including the top 15 variables was sufficient to achieve optimal predictive accuracy.

Using the Gini index, 15 VOCs were selected based on their contribution to class discrimination, leading to improved model performance.

**Figure 2:**
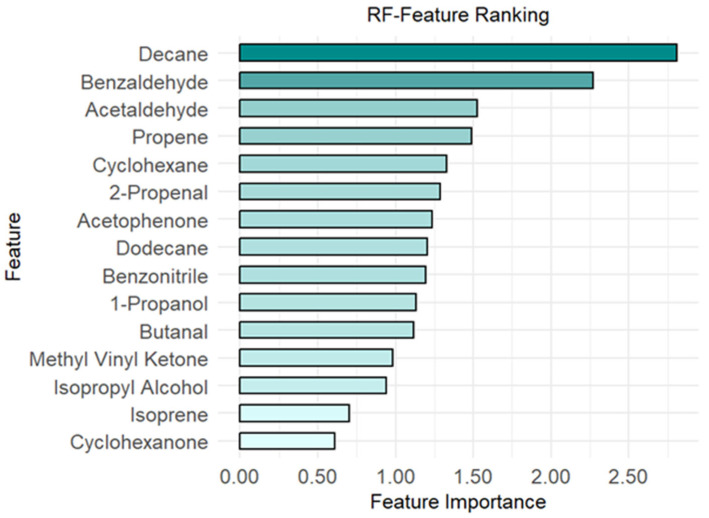
The first 15 ranked features of the Random Forest (RF) classifier.

More specifically, as illustrated in Figure 2, the compounds identified as potentially diagnostic include decane, benzaldehyde, acetaldehyde, propene, 2-propenal, cyclohexane, acetophenone, dodecane, benzonitrile, 1-propanol, butanal, chlorobenzene, methyl vinyl ketone, isopropyl alcohol, isoprene, and cyclohexanone.

**Figure 3:**
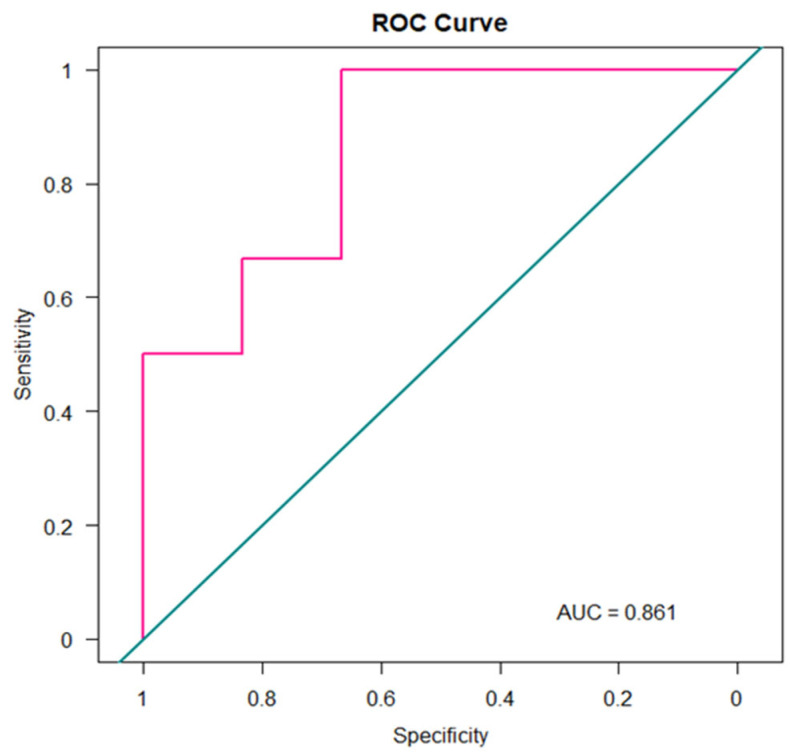
ROC Curve.

The final RF classifier achieved an average classification accuracy of 80%, indicating good discriminative performance across the analyzed conditions. Furthermore, to assess the model’s diagnostic performance in distinguishing MPM patients from healthy controls (HC), a receiver operating characteristic (ROC) curve was constructed. ROC analysis was performed using the pROC package in R (version 3.5.1), providing a quantitative measure of the model’s discriminative power. As shown in Figure 3, the resulting area under the curve (AUC) was 0.86, highlighting the model’s potential clinical utility.

**Figure 4:**
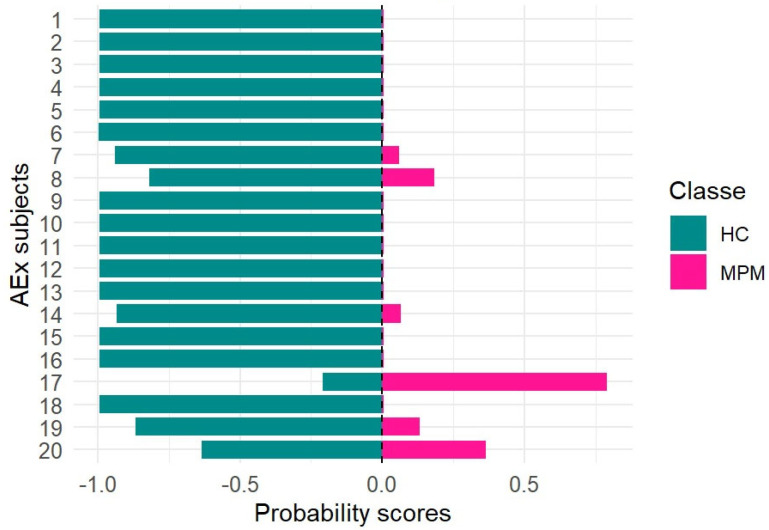
Probability scores for at-risk asbestos-exposed subjects (AEx).

In order to evaluate the performance of the model developed and validated in this study for the screening of MPM and management of at-risk asbestos-exposed subjects, the random forest (RF) model was applied to a dataset consisting of breath samples collected from asbestos-exposed individuals (EXP). More specifically, 20 samples were analyzed and processed as blinded inputs by the trained model. The probabilities of belonging to the MPM or HC class for each asbestos-exposed subject were obtained and reported in Figure 4.

**Figure 5:**
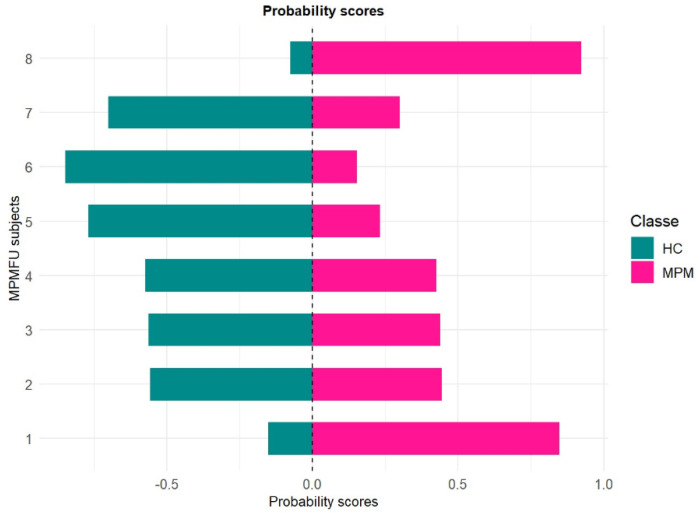
Probability scores for MPMFU subjects.

The statistical results obtained in this study using a larger sample size are consistent with those reported in our previous work [5]. More specifically, almost all blinded samples classified as controls by the model were confirmed to be healthy following further diagnostic procedures, such as CT scans. Only breath sample no. 17, which was classified as pathological with a probability score of 0.8, was subsequently diagnosed with malignant pleural mesothelioma (MPM) based on CT findings. The remaining five breath samples, showing probability scores below 0.5, were likely associated with a pre-cancerous condition, as CT scans revealed the presence of pleural plaques. If these results were confirmed in a study conducted on a larger population, the early identification of pre-cancerous lesions—given their potential to evolve into MPM over time—would allow closer monitoring of at-risk subjects through a more accurate and frequent follow-up. Finally, in order to assess the potential applicability of the random forest (RF) model developed in this study for monitoring malignant pleural mesothelioma patients during follow-up (MPMFU), an exploratory analysis was performed on an independent set of samples. Specifically, eight breath samples collected from patients initially enrolled at baseline were analyzed after completing their chemotherapy cycle and processed as blinded samples. The results obtained for each blinded sample, using the RF model trained on the MPM and HC classes, were expressed as probabilities of belonging to either class. These probabilities provided insights into potential disease progression (Figure 5).

As shown in Figure 5, the application of the random forest model to the blinded samples yielded a heterogeneous probability profile among the MPMFU subjects. Two patients (n.1 and 8) exhibited higher probability scores (higher than 0.5) for the MPM class, indicating an increased likelihood of belonging to the pathological group. Breath sample n. 8 was classified with a high probability of MPM affiliation, while all the other individuals exhibited more intermediate profiles, suggesting a less advanced disease state. This interpretation is supported by follow-up CT scan data obtained post-therapeutic intervention. In fact, patients no. 1 and no. 8 were found to be in a state of disease progression, while all others showed stable disease after therapy. As part of an exploratory investigation, these preliminary findings suggest that the model may offer a valuable probabilistic index for longitudinal monitoring of patients. However, further validation in larger and independent cohorts is required to confirm its clinical utility.

## 3. Discussion

In this study, the statistical treatment of experimental data enabled the identification of a pattern of VOCs capable of discriminating between MPM patients and healthy controls with 86% accuracy. More specifically, as reported in Figure 2, the most important VOCs identified as potential diagnostic markers included alkanes, alkenes, aldehydes, ketones, alcohols, and aromatic derivatives. Nowadays, a validated list of VOC biomarkers for MPM has not yet been identified in human breath. This knowledge gap arises from several factors, including the lack of standardized protocols for breath sampling and analysis, the differences in study design (such as control group selection and patient recruitment criteria), the size of the study population, and the variations in data-mining approaches [30,31,32,33]. Although these factors could affect breath analysis outcomes, the variables found in this study as discriminating between the MPM and HC groups are consistent with those previously reported in the scientific literature as biomarkers of MPM or lung cancer sharing pathophysiological similarities [5,6,24,27,28,30,34,35,36,37,38,39,40]. More specifically, in this study, 15 VOCs, including decane, benzaldehyde, acetaldehyde, propene, 2-propenal, cyclohexane, acetophenone, dodecane, benzonitrile, 1-propanol, butanal, chlorobenzene, methyl vinyl ketone, isopropyl alcohol, isoprene, and cyclohexanone, were identified as potential biomarkers.

Decane has been identified in exhaled breath as a potential biomarker in various pathological conditions, including malignant pleural mesothelioma (MPM) and lung cancer. Its presence in breath samples, pleural effusions, and in vitro lung cancer cell lines has been associated with oxidative stress and lipid peroxidation, processes driven by the production of oxygen-derived free radicals [26,41,42,43]. Moreover, decane has also been detected as a potential biomarker of occupational exposure in environments such as metal smelting and fire-fighting environments, further supporting the hypothesis that its occurrence may be linked to chronic inflammation induced by environmental contaminants [30,44]. The generation of decane is thought to result from the degradation of polyunsaturated fatty acids (PUFAs) mediated by reactive oxygen species (ROS), thereby reflecting the broader contribution to the volatile organic compound (VOC) profile associated with oxidative damage, a condition frequently present in cancer. Similarly, dodecane is believed to originate from lipid peroxidation and has been detected in samples from patients with both lung cancer and asthma, supporting an inflammatory basis for its occurrence [45]. Specifically, it has been proposed to result from the degradation of alkoxyl radicals into alkyl radicals, which subsequently stabilize into saturated hydrocarbons [46,47].

Benzaldehyde, although frequently regarded as an exogenous volatile organic compound (VOC), has been consistently detected in human breath and in both in vivo and in vitro studies as part of a VOC signature capable of discriminating between malignant pleural mesothelioma (MPM) and healthy controls (HC), as well as between occupationally exposed and unexposed individuals [28,48]. Additionally, several studies have identified benzaldehyde in the breath of lung cancer patients [49,50,51], as well as in other pathological conditions such as chronic kidney disease, suggesting its potential involvement in shared metabolic pathways across different diseases or its association with persistent environmental exposures [52]. Lipid hydroperoxides and aldehydes generated during lipid peroxidation may enhance benzaldehyde production via interactions with amino acids. One study examined the degradation of phenylalanine in the presence of linoleic acid 13-hydroperoxide and 4-oxo-nonenal, demonstrating that the combined action of reactive carbonyl species and free radicals promotes the formation of benzaldehyde from phenylalanine [53]. It is also worth noting that endogenous benzaldehyde may derive from benzyl acetate, benzoic acid, and benzyl alcohol, which are derived from dietary intake because they are commonly used as flavoring agents, food additives, and preservatives. In fact, benzyl acetate is rapidly hydrolyzed in the body to form acetic acid and benzyl alcohol, which is then oxidized to benzaldehyde by alcohol dehydrogenases. Under physiological conditions, these compounds are efficiently metabolized by hepatic enzymes and excreted via urine. However, cancer-related metabolic dysregulation may impair this clearance, potentially leading to an accumulation of benzaldehyde in affected individuals. In addition to benzaldehyde, several studies have detected acetaldehyde in the exhaled breath of oncological patients as well as in the headspace of lung cancer cell cultures [51,53,54,55,56]. In particular, in vitro experiments have demonstrated that non-small cell lung cancer (NSCLC) cell lines release acetaldehyde in amounts proportional to cell number. This observation suggests a potential correlation between acetaldehyde concentration and tumor size, indicating that, in vivo, breath levels of this compound may reflect cancer progression [57,58]. All these findings are likely attributable to altered mitochondrial function and metabolic reprogramming consistent with the Warburg effect. Moreover, acetaldehyde may be endogenously produced from ethanol via the action of alcohol dehydrogenase 1B (ADH1B), which catalyzes its oxidation via the hepatic alcohol metabolic pathway. In physiological conditions, acetaldehyde is then metabolized to acetate by mitochondrial aldehyde dehydrogenase 2 (ALDH2) [59,60], but genetic polymorphisms in human ALDH2 and ADH1B associated with cancer could result in the rapid accumulation of acetaldehyde, which can cross biological membranes, enter the bloodstream, and be excreted via breath [61,62]. Another aldehyde showing altered levels in the breath of lung cancer patients is 2-propenal, commonly known as acrolein. Specifically, several studies have reported decreased concentrations of this compound in both in vitro and in vivo models [50,54,63,64]. Given its high reactivity and known cytotoxic properties, such a reduction may reflect shifts in cellular metabolism or detoxification mechanisms associated with malignancy. Its involvement in pathological processes was initially proposed following the identification of the acrolein–lysine adduct (FDP-lysine) in oxidized low-density lipoproteins (LDL) in vivo, suggesting a potential role for this aldehyde in the pathogenesis of several oxidative stress-related diseases. Although its precise role in cancer-related VOC profiles remains unclear, the consistent alterations observed across studies support its relevance as a potential marker of disrupted redox homeostasis [65], reflecting alterations in the lipid peroxidation of polyunsaturated fatty acids, such as α-linolenic acid and eicosatrienoic acid, or potentially originating from bacterial catabolism of amino acids like leucine, as well as from intermediary metabolic pathways induced by neoplastic processes [66,67].

Propene, an unsaturated hydrocarbon, has been identified as a potential biomarker in several studies; however, its interpretation remains challenging [63]. Elevated levels have been observed in smokers, suggesting a predominantly exogenous origin related to cigarette smoke [44]. Moreover, the detection of propene in breath profiles associated with bronchial infections and cystic fibrosis may further suggest a link with respiratory infections and chronic inflammation [64]. Anyway, no well-defined endogenous biosynthetic pathway regarding propene has been identified in humans. Conversely, isoprene is among the most extensively studied VOCs due to its high abundance in human breath. Although traditionally associated with cholesterol biosynthesis via the mevalonate pathway in the liver, recent evidence indicates that its production predominantly arises from muscle lipolytic metabolism of cholesterol [68,69]. Furthermore, radical peroxidation of polyunsaturated fatty acids (PUFAs) under cancer-related conditions characterized by chronic inflammation and elevated reactive oxygen species (ROS) production may contribute to increased isoprene levels in blood and breath [70]. However, isoprene levels exhibit considerable inter-individual variability and are influenced by physiological factors such as age, sex, ventilation, pulmonary perfusion, and physical activity [40]. Despite its detection in breath of patients with MPM and other oncological conditions, its diagnostic utility is limited by this variability, its susceptibility to extrinsic factors, and sampling protocols, thereby reducing its reliability as a standalone biomarker [30,51]. Another alkane recognized as a biomarker of lung cancer and malignant pleural mesothelioma in studies focused on breath analysis is cyclohexane [21,28]. More specifically, cyclohexane levels in breath samples have been reported to be stage-dependent, with a reduction observed in small cell lung cancer (SCLC) following effective therapeutic intervention [15,71]. Notably, cyclohexane production has been associated with oxidative stress, inflammatory processes, and the degradation of ε-caprolactam, suggesting potential mechanistic links between xenobiotic metabolism and carcinogenesis [28]. Moreover, the oxidation of cyclohexane via endogenous biochemical pathways may lead to the formation of cyclohexanone, which was also identified in this study as a biomarker of MPM. In several studies involving both breath analysis and in vitro investigations, acetophenone and methyl vinyl ketone have also been identified as a discriminating marker between malignant pleural mesothelioma (MPM) and healthy control (HC) groups as well as among MPM histological subtypes, including biphasic, epithelioid, and sarcomatoid variants [5,28,50,51,72,73,74,75]. Acetophenone is an intermediate in the human metabolic pathway of ethylbenzene degradation. Under conditions of pronounced oxidative stress, such as those occurring in certain oncological diseases, a functional impairment of phenylalanine hydroxylase (PAH) has been reported. This enzymatic deficit impedes the normal conversion of L-phenylalanine into tyrosine, redirecting excess phenylalanine into alternative metabolic routes that generate phenylketones, including acetophenone [5]. Methyl vinyl ketone, in contrast, is likely generated through the oxidative degradation of fatty acids or as a secondary product of lipid peroxidation, thereby indicating a broader profile associated with oxidative stress [76]. Other compounds detected in breath samples and likely associated with oxidative stress include alcohols, particularly 1-propanol, which has been frequently reported in the literature as a potential biomarker for MPM and non-small cell lung cancer (NSCLC) [29,38,49,55,64,76,77]. Furthermore, it has been suggested that 1-propanol detected in blood and subsequently in breath samples may result from altered NAD^+^/NADH ratios in cancer cells, specifically linked to the overexpression of alcohol dehydrogenase (ADH) in tumor tissues [78,79].

Finally, although benzonitrile and chlorobenzene have been identified as potential VOC markers of malignant pleural mesothelioma (MPM) and lung cancer in several studies, the underlying metabolic or cancer-associated catabolic pathways responsible for their presence in the breath of MPM patients remain poorly understood [5,44,80]. Further investigations are required to elucidate the biochemical mechanisms leading to their production and accumulation.

Overall, the consistent detection of these VOCs across diagnostic studies underscores their relevance as biomarkers and highlights the convergence of metabolic pathways—particularly those involving oxidative stress, lipid peroxidation, and inflammation—in cancer pathophysiology.

## 4. Materials and Methods

### 4.1. Experimental Study Design

A multicenter cross-sectional observational study approved by the Local Ethics Committee (Prot. n. 22 of 14/01/2019) was carried out from 2021 to 2024 in accordance with the Declaration of Helsinki and thanks to the collaboration between the research group of the one Health Laboratory of the Department of Biosciences, Biotechnologies and Environment of University of Bari and the medical team of the Thoracic Oncology Unit of the Cancer Institute of Bari. A total of 125 volunteers aged from 39 to 84 years, including 61 healthy controls (HC group) and 64 patients with malignant pleural mesothelioma (MPM group), were enrolled in the study. All non-hospitalized volunteers were selected after a computed tomography (CT) scan or chest radiograph confirming either a healthy or pathological condition, and before starting pharmacological treatment in patients with advanced MPM. Persons underage, unable to provide a breath sample, or affected by pathological conditions such as malignant neoplasms, liver disease, asthma, chronic obstructive pulmonary disease, or upper or lower respiratory tract infections, were not included in the study. Comprehensive biographical and clinical data, including information on concomitant disorders, medications, habits, such as smoking behavior and previous asbestos exposure, were collected from all participants and reported in Table 1. Taking into account that MPM is not associated with prior smoking habits, smoking was not considered a confounding factor [81,82]. Each participant signed an informed consent form and abstained from food, beverages, and smoking for at least 12 h before breath sampling. As shown in Table 1, the two volunteer groups (HC and MPM) were comparable regarding age, body mass index (BMI), gender, and clinical status.

Moreover, in order to evaluate the potential of breath analysis in the screening of MPM and management of at-risk individuals, 20 healthy subjects exposed to asbestos were also enrolled in the study. Finally, aiming to explore the clinical utility of breath analysis for the monitoring of disease progression during follow-up of patients, among the recruited MPM patients, the exhaled breath samples of 8 subjects in therapeutic follow-up (MPMFU) were collected about 6 months later at the end of 4–6 rounds of chemotherapy.

### 4.2. Breath Sampling and Analysis

In this study, end-tidal breath samples were collected by the standardized method reported by Di Gilio et al., 2024 and 2020 [52,83] inside a dedicated outpatient of the Apulian Regional Centre for Breath Analysis where no disinfectant agents or other potentially contaminating products were used. Moreover, an ambient air (AA) sample was collected inside the dedicated ambulatory and at the same time as breath sampling in order to exclude environmental VOCs potentially affecting breath samples. Both the end-tidal fraction of the exhaled breath and the AA samples were collected by using an automated sampler named "Mistral," a medical device developed by an R&D company named Predict s.r.l. (Bari, Italy) with scientific support of the research group of the OneHealth Laboratory of the Biosciences, Biotechnologies and Environment Department of the University of Bari.

Based on a standardized protocol, the volunteers remained at rest for at least 10 min before the breath collection in order to guarantee the achievement of equilibrium between the lungs and ambient air. During this period, all the sampling lines inside the automatic sampler were purged with 1 l of ambient air. Potential contamination linked to device components and materials has been exhaustively evaluated in our previous studies [36]. Once the device cleaning was finished and the AA sample collected, the volunteers breathed calmly through a mouthpiece and slowly exhaled the full expiratory vital capacity. A wide range of VOCs (C3–C20) in AA and in the end-tidal fraction of the exhaled breath were separately collected onto suitable two-bed sorbent tubes packed with Tenax TA and Carbograph 5 TD (Bio-monitoring steel tube, Markes International Ltd., Bridgend, UK).

The collected samples were subsequently analyzed using a thermal desorber (UNITY-xr—Markes International Ltd., UK) coupled with a gas chromatograph (GC 7890, Agilent Technologies Inc., Santa Clara, CA, USA) and a mass spectrometer (MS 5975, Agilent Technologies Inc., Santa Clara, CA, USA). An optimized analytical methodology is reported in our previous study [5]. Briefly, VOCs adsorbed onto the cartridges were thermally desorbed in splitless mode at 300 °C for 10 min, refocused at 20 °C onto a cold trap. A cold trap specific for wet samples (U-T4WMT-2S Water Management, Markes International Ltd., Llantrisant, UK) was used to concentrate organic compounds between ethane and C20 in a narrow band at the head of a 5% diphenyl/95% dimethyl polysiloxane capillary column (60 m × 250 µm × 0.25 µm film thickness-VOCOL^®^-Supelco, Merck KGaA, Darmstadt, Germany). A customized mix standard solution containing 44 VOCs at a concentration of 10 µg/mL in methanol (Ultra Scientific Cus-5997, ULTRA Scientific Italia s.r.l., Bologna, Italy) was daily analyzed to verify the response factors of the GC/MS system over time. Single target ions were extracted from TIC chromatograms (Extracted Ions Chromatograms, EIC mode) using GC/MS post-run analysis software (Agilent Mass Hunter Qualitative Analysis-Agilent Technologies Ltd., Santa Clara, CA, USA), and a semi-quantitative analysis was performed in order to consider not only the VOCs included in the standard solution. Therefore, VOC identification was based on the comparison of retention times and ion ratios between VOCs in breath samples and those in the standard solution. For the remaining VOCs not included in the standard solution, the identification involved the comparison between the obtained mass spectrum and those included in the National Institute of Standards and Technology library (2017), considering a matching higher than 80%. A total of 98 chromatographic peaks with an intensity higher than five times the baseline signal and not linked to device components and materials contamination were integrated, and the corresponding areas were included in the dataset.

### 4.3. Data Analysis

**Figure 6:**
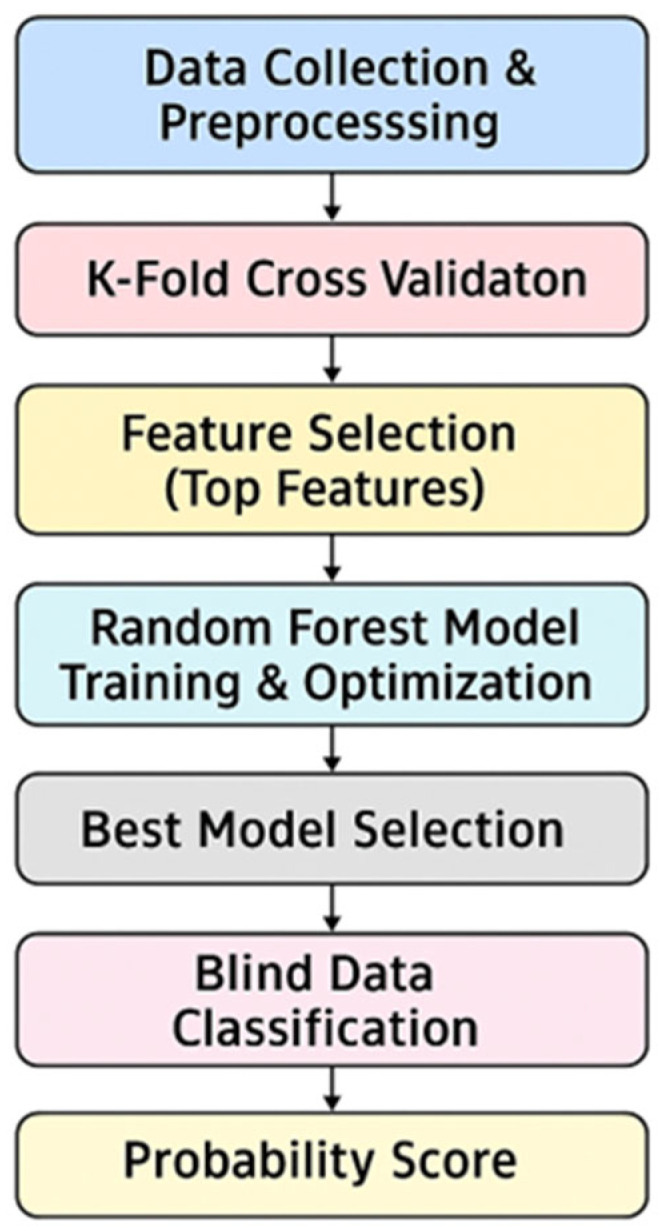
The machine learning workflow.

Statistical data analysis was performed using the R Studio interface, version 4.5.0 (R Foundation for Statistical Computing, Vienna, Austria), using a data matrix consisting of the peak area of the 98 VOCs detected in breath samples. Experimental data were statistically processed using a machine learning approach based on a random forest algorithm. The model was trained using a k-fold cross-validation framework and by dividing the entire dataset into ten balanced subgroups (with k = 10) to ensure robust performance evaluation. In each iteration round, nine subgroups were used for training, while the tenth served as a test. This strategy ensured a reliable estimate of the predictive capacity of the model, reducing the risk of overfitting. Feature selection for the RF classifier was carried out using an embedded method based on the Gini index. Starting from this ranking, models were iteratively built using progressively larger subsets of variables in order to identify the optimal number of compounds to achieve the best classification performance. Model performance was evaluated using ROC analysis and considering the area under the ROC curve (AUC) as a global indicator of classification accuracy. Finally, the model was used to assign a predictive score to the subjects at-risk for asbestos exposure (EXP) and in follow-up (MPM) processed as blinded samples in order to evaluate the probability of belonging of each sample to the MPM or HC class. The machine learning workflow employed in this study is illustrated in Figure 6.

## 5. Conclusions

Breath analysis combined with machine learning-based classification confirms its potential as a valuable tool for the early diagnosis of malignant pleural mesothelioma (MPM). Considering the rarity of MPM and the inherent challenges in recruiting affected patients, this study developed and validated a robust data mining approach on a broader dataset than those used in previously published research.

More specifically, in this study, a pattern of VOCs—including alkanes, alkenes, aldehydes, ketones, alcohols, and aromatic derivatives—was able to discriminate between MPM patients and healthy controls with a classification accuracy of 86%. The identified VOCs, in line with the scientific literature, reflect key metabolic alterations characteristic of MPM pathophysiology, such as oxidative stress, membrane lipid peroxidation, and aberrant enzymatic activity within tumor tissues.

The explorative application of the developed random forest classification model to blinded breath samples exhaled from at-risk asbestos exposure subjects and MPM patients during follow-up yielded probability scores in alignment with clinical outcomes, supporting the feasibility of breath-based longitudinal monitoring.

Nevertheless, despite the promising and literature-consistent results obtained in this study, the limited size of the study population, particularly among at-risk individuals and MPMFU patients, warrants further investigation. More specifically, validation in larger and independent cohorts is essential to confirm the specificity, sensitivity, and overall robustness of this approach, ultimately improving early detection, prognosis, and therapeutic strategies for MPM patients. Therefore, thanks to the establishment of the first Apulian Breath Analysis Center in southern Italy, the collection of breath samples from patients in follow-up and at-risk individuals is currently ongoing. This effort aims to confirm and validate our current findings within the framework of a prospective clinical study.

## Figures and Tables

**Table 1:** Characteristics of the study population.

Variation	HC	MPM	AEx	MPMFU
Subject	61	64	20	8
Median Age (Years)	59 (39–81)	69 (50–84)	66.6 (56–81)	70.25 (58–76)
Male/Female	39/22	42/22	14/6	4/4
Median BMI (Kg/m^2^)	23.42	24.90	23.7	22.87
Smokers	11	5	9	1
Ex-smokers	17	23	5	3
Non smokers	33	36	6	4
Diabetes	1	4	5	2
Hypertension	19	27	7	4
Hypercholesterolemia	9	12	5	2

## Data Availability

The data presented in this study are available on request from the corresponding author (ADG) due to privacy and ethical reasons.
